# Detection and risk stratification of women at high risk of preterm birth in rural communities near Nagpur, India

**DOI:** 10.1186/s12884-017-1504-4

**Published:** 2017-09-19

**Authors:** Archana Patel, Amber Abhijeet Prakash, Yamini V. Pusdekar, Hemant Kulkarni, Patricia Hibberd

**Affiliations:** 1 Lata Medical Research Foundation, Nagpur, India; 2 South Texas Diabetes and Obesity Institute, University of Texas Rio Grande Valley, Brownsville, TX 78520 USA; 3 Department of Global Health, Boston University School of Public Health, Boston, MA USA

**Keywords:** Preterm birth, Risk stratification, Diagnostic accuracy, Community workers

## Abstract

**Background:**

Presently, preterm birth is globally the leading cause of neonatal mortality. Prompt community based identification of women at high risk for preterm births (HRPB) can either help to avert preterm births or avail effective interventions to reduce neonatal mortality due to preterm births. We evaluated the performance of a package to train community workers to detect the presence of signs or symptoms of HRPB.

**Methods:**

Pregnant women enrolled in the intervention arm of a cluster randomized trial of Antenatal Corticosteroids (ACT Trial) conducted at Nagpur, India were informed about 4 directly observable signs and symptoms of preterm labor. Community health workers actively monitored these women from 24 to 36 weeks of gestation for these signs or symptoms. If they were present (HRPB positive) the identified women were brought to government health facilities for assessment and management. HRPB positive could also be determined by the provider if the woman presented directly to the facility. Risk stratification was based on the number of signs or symptoms present. The outcome of preterm birth was based on the clinical assessment of gestational age < 37 weeks at delivery or a birth weight of <2000 g.

**Results:**

Between July 1, 2012 and 30 November, 2013, 686 of 7050 (9.7%) pregnant women studied, delivered preterm. 732 (10.4%) women were HRPB positive, of whom 333 (45.5%) delivered preterm. Of the remaining 6318(89.6%) HRPB negative women 353 (5.6%) delivered preterm. The likelihood ratio (LR) of a preterm birth in the HRPB positives was 8.14 (95% confidence interval 7.16–9.26). The LR of a preterm birth increased in women who had more signs or symptoms of HRBP (*p* < 0.00001). More signs or symptoms of HRPB were also associated with a shorter time to delivery, lower birth weight and higher rates of stillbirths, neonatal deaths and postnatal complications. Addition of risk stratification improved the prediction of preterm delivery (Integrated Discrimination Improvement 17% (95% CI 15–19%)).

**Conclusions:**

The package for detection of signs and symptoms of HRPB is feasible, promising and likely to improve management of preterm labor.

**Trial registration:**

NCT01073475 on February 21, 2010 and NCT01084096 on March 9, 2010.

## Background

The World Health Organization estimates that every year approximately 15 million babies are born prematurely and 1 million of these babies die despite available interventions that could save 75% of them [1]. Globally, preterm birth is increasing and is now the leading cause of death in neonates and children under age 5 [2]. In addition to mortality, the burden of preterm birth results from significant lifetime disability in survivors, accounting for 3.1% of all Disability Adjusted Life Years (DALYs) [3, 4]. This burden of disease is primarily due to breathing problems, feeding difficulties and neurological problems, with effects reaching into adulthood [5].

The vast majority (85%) of preterm births occur in low and lower middle income countries in Asia and Africa [5]. India has the highest number of preterm births in the world accounting for 23.6% of the global burden with approximately 1 in 8 babies being preterm [1]. It is difficult to recognize preterm labor in under-equipped, lower level facilities since access to ultrasound assessments and measurement of biochemical markers such as fetal fibronectin is limited in these settings [6]. Failure to identify women at high risk for preterm birth in rural communities, delays access and utilization of interventions that may prevent preterm deliveries.

Currently the standard of care for pregnant women at high risk of preterm birth (HRPB) is administration of antenatal corticosteroids at least 48 h before delivery [7, 8]. In a systematic review based on 18 trials in resource rich countries, antenatal corticosteroids resulted in a 34% reduction in the incidence of RDS, a 46% reduction in intraventricular hemorrhage and a 31% reduction in neonatal mortality [9]. In resource limited settings, antenatal corticosteroids are available only at tertiary health care facilities that can administer antenatal corticosteroids and offer other specialized perinatal interventions that improve preterm outcomes and is rarely accessible to pregnant women in rural communities. Moreover, the common causes of preterm births include anemia, maternal infections, antepartum hemorrhage and pregnancy induced hypertension [2]. Thus early identification of HRPB women can also have additional benefits in terms of recognition of these other obstetric conditions associated with preterm births. Therefore, there is an urgent need to identify pregnant women at HRPB well before delivery.

We recently participated in a multi-site cluster randomized trial conducted by the Global Network for Women and Children’s Health Research [10]. This trial (the ACT Trial) evaluated a multifaceted intervention for detection of women at HRPB so that they could receive antenatal corticosteroids with a goal of improving neonatal outcomes. Accuracy of how well this intervention performed to predict preterm births was evaluated using the data from the ACT trial. Furthermore we evaluated whether risk stratification based on number of symptoms present, improved prediction of delivering a preterm baby.

## Methods

### Study design, setting, and ethics statement

This study is a secondary data analysis using data from the intervention arm of the ACT Trial [10, 11] and Maternal and Newborn Health (MNH) Registry [12] supported by the Eunice Kennedy Shriver National Institute of Child Health and Human Development NICHD’s Global Network for Women’s and Health Research. For this study, we used the data collected at the Nagpur, India site of these multicenter studies.

For the MNH Registry, a prospective cohort of pregnant women was recruited as early as possible during pregnancy and followed through day 42 (up to a maximum of 60 days) post-partum to obtain details about the pregnancy, labor, delivery and the health of the mother and infant. In Nagpur, pregnant women were recruited consecutively from the rural communities in the catchment areas of 20 Primary Health Centers (PHCs) or clusters in 4 districts – Nagpur, Bhandara, Wardha and Chandrapur. In the ACT trial, ten of the 20 PHCs were randomized to the multifaceted ACT intervention arm between June 11, 2012 and December 11, 2013. Study variables for the ACT trial were collected in the MNH Registry, supplemented by additional data collection in the ACT intervention clusters. Most babies (99.02%) were born in health facilities as a result of *Janani Suraksha Yojana* (JSY) that provides financial assistance for woman to promote institutional deliveries and to ASHAs (Accredited Social Health Activist) who bring the expectant mother to institutions for delivery. This scheme has substantially increased rates of deliveries at facilities in India [13].

### Participants

**Fig. 1:**
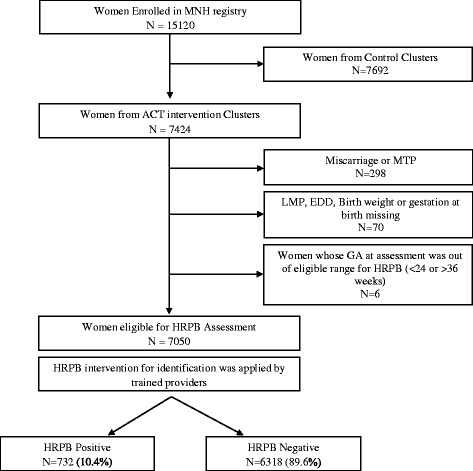
Flow diagram of study participants

We included women enrolled in the MNH Registry before week 24 of pregnancy (Fig. 1) and excluded those enrolled in the ACT trial control clusters because there was no detection of HRPB in the control clusters. Additional exclusion criteria for this secondary data analysis included: i) miscarriage or medical termination of pregnancy; ii) no information about gestational age prior to delivery; and iii) missing birth weight.

### Definition and risk stratification of HRPB

Women were classified as HRPB positive if they had one or more of the 4 following signs or symptoms of preterm labor during the risk period of week 24–36 of gestation: (1) intermittent abdominal pain or pain associated with blood-stained mucus discharge, watery vaginal discharge or a sudden gush of water (Signs of preterm labor); (2) watery vaginal discharge or a sudden gush of water with or without cramping (Premature rupture of membranes); (3) vaginal bleeding with or without cramping (hemorrhage), and (4) severe headache (hypertension) [10]. Risk of preterm birth was further stratified by the number of signs or symptoms present ranging from 0, 1, 2 or 3 or more. The HRPB definition did not include a history of previous preterm birth, a well-recognized predictor of preterm birth, [2, 14] because of the difficulty in obtaining a reliable history of previous spontaneous preterm birth in rural settings [10].

### Intervention package to determine HRPB

The multifaceted HRPB package consisted of four components – i) training at multiple levels regarding signs or symptoms of preterm labor, ii) monitoring in the community between week 24 and 36 of gestation by community health worker or ASHA worker, iii) determination of whether the pregnant woman was positive using the HRPB signs or symptoms by ASHA worker in the community, and, iv) confirmation of the HRPB signs or symptoms by a health care provider (HCP) at the Primary Health Center (PHC), sub-center (SC) or other referral facility to determine eligibility for administration of ACT in a woman who is brought to the center by an ASHA or who is self referred.

### Multi-level training for signs or symptoms to detect HRPB

Awareness regarding the 4 signs or symptoms of preterm labor during the risk period (week 24–36 of gestation) was created in the community and in households with pregnant women. The pregnant women also received pamphlets containing these details as reminders. The ASHA workers who live in the community and are responsible for about 10 pregnant women were trained by study obstetricians to ask and recognize the signs or symptoms and record positive responses on the women’s tracking card (the Nagpur based study staff informed the ASHA workers when the risk period started and ended i.e. week 24–36 week of gestation). Training started with a pre-training questionnaire to assess baseline knowledge about preterm birth, its consequences and how to identify women who might be at HRPB. It was followed by audio-visual presentations supplemented by posters, pamphlets and manuals translated into the local language (Marathi), role-playing with qualified obstetricians, and learning how to use an obstetric disc and a color coded tape to assess fundal height and estimate gestational age (see [10] for details of the use of tape). They were also trained to track the pregnant women during the risk period by conducting weekly home visits, to ask about signs or symptoms of HRPB and to record positive responses on the woman’s tracking card and to record. The HCPs in 10 PHCs and their attached SCs (first level facilities) and 15 referral facilities were trained by study obstetricians to recognize the signs or symptoms of HRPB in a similar way to the ASHA workers, as described above. They were retrained every 6 months.

### Monitoring, determination and confirmation of HRPB

The ASHA tracked each pregnant woman by conducting weekly home visits during the risk period and recorded positive HRPB signs or symptoms on tracking cards. They brought women who were positive for HRPB to a health facility which could be the SC or PHC or a referral facility. The HCP would confirm HRPB positivity and administer ACT. Women could also directly reach the health facilities, if they suspected that they felt that they were at high risk for preterm birth. The HCP were the auxiliary nurse midwives (ANMs) at SCs, the medical officers at the PHC, and the nurses, residents and obstetricians at the referral facilities. Each pregnant woman could be evaluated multiple times if she reported signs or symptoms at different times, but she could be HRPB positive only once.

### Primary outcome - preterm birth

The primary outcome of interest was whether the pregnant women delivered a preterm or term baby based on the reported gestational age at birth and birth weight from the MNH registry. The medical officers at the place of delivery routinely use a combination of the following criteria to report gestational age at birth: 1) date of last menstrual period if menses are regular, 2) first trimester ultrasound if available, 3) birth weight 4) clinical evaluation of the neonate. Based on these reports, a baby was considered preterm if the reported gestational age was <37 weeks. Birth weight < 2000 g (5th percentile of birth weight) was also considered a preterm birth. The GN study used birthweight rather than gestational age for the primary analysis subgroup because many women in the registry had missing or uncertain gestational age, ultrasound was often unavailable, and the intervention was designed to improve estimation of gestational age, which could potentially bias gestational age-based analyses [10].

### Data source and assessment

Trained study staff collected data for the MNH Registry at three time points. The first time point was on enrolment (before the 20th week of gestation whenever possible), during which information on the date of last menstrual period, estimated delivery date, age, education, parity, and status of their last child is collected. The second time point was within 7 days of delivery, information is collected on prenatal care, birth preparedness, complications occurring during pregnancy, details of labor and delivery, including place, mode of delivery, provider, actual birth weight obtained at the time of birth, status of the mother and newborn (including term or preterm as noted by the facility physician or specialist) following delivery, referrals, and treatment provided to the mother and newborn at referral facilities. The third time was 42 (up to a maximum of 60) days after birth. Separately trained ACT trial study staff tracked women who were HRPB positive from place of identification to the facility where they received the antenatal corticosteroids and delivered.

### Statistical analysis

Performance of the package of intervention to identify HRPB women was assessed using likelihood ratios (LR) for each risk category. Likelihood ratio was defined as the ratio of post-test probability and prevalence of preterm birth for each risk category. We used methods described by Centor [15] to estimate the LRs and their 95% confidence intervals (CI). To estimate the independent contribution of HRPB risk stratification for the identification of preterm births, we used multivariable generalized estimating equations (GEE) to account for inter-cluster variation and the following covariates: mother’s age, mother’s education, parity, and presence of anemia. Dose-response was assessed using the Cochrane-Armitage test for linear trend and category-specific probability of a preterm birth. Improvement in the prediction of a preterm birth based on the package of intervention was evaluated using the integrated discrimination improvement (IDI) and the net reclassification index (NRI) [16]. We used the IDI and NRI packages (http://personalpages.manchester.ac.uk/staff/mark.lunt) to determine the IDI and NRI values (continuous version without using cut-offs), respectively. These programs directly compare the discrimination and reclassification ability of a new predictor over and beyond the predictors included in the baseline model.

Finally, we explored whether there were associations between increasing number of signs or symptoms of HRPB and time to delivery, birth weight and neonatal outcomes using the Cochrane-Armitage test for linear trend. Data were analyzed using Stata 13.1 package (Stata Corp, College Station, Texas, USA). Statistical significance was tested with two-tailed tests and a type I error rate of 0.05.

## Results

Of the women enrolled in the ACT trial between July 1, 2012 and 30 November, 2013, a total of 7050 pregnant women were included in the final analysis of this study (Fig. 1). Table 1 shows the characteristics of the women. The majority were between age 20 and 25 years, had secondary or higher educational background, and were anemic. Forty-five percent were nulliparous. In the parous women, 96% women had a history of previous live birth. Presence of signs or symptoms of HRPB was first made by the ASHA workers for 73% of the women diagnosed as HRPB, with an additional 22% being identified at a PHC and the remaining 5% identified at a referral hospital.

**Table 1:** Characteristics of the Study Population (*N* = 7050)

Characteristic	Preterm	Term	Total
	*N* = 732 (100)	*N* = 6318 (100)	7050 (100)
Mother’s Age			
<20	17 (2.32)	99 (1.57)	116 (1.65)
20–25	570 (77.87)	4833 (76.5)	5403 (76.64)
>25	145 (19.81)	1386 (21.94)	1531 (21.72)
Mother’s Education^a^			
None-Primary	172 (23.5)	1361 (21.54)	1533 (21.74)
Secondary	456 (62.3)	3792 (60.02)	4248 (60.26)
University	104 (14.21)	1152 (18.23)	1256 (17.82)
Parity			
Nulliparous	366 (50)	2828 (44.76)	3194 (45.3)
1–2	344 (46.99)	3325 (52.63)	3669 (52.04)
>2	22 (3.01)	165 (2.61)	187 (2.65)
Previous birth live (for parity ≥1)			
Yes	343 (93.7)	3354 (96.1)	3697 (95.9)
No	23 (6.28)	136 (3.9)	159 (4.1)
Maternal Anemia (Hemoglobin g%)^a^			
Not anemic (≥11)	36 (4.92)	506 (8.01)	542 (7.69)
Mild (10–11)	262 (35.79)	2636 (41.72)	2898 (41.11)
Moderate/ Severe (<10)	434 (59.29)	3143 (49.75)	3577 (50.7)
Hypertension			
Yes	132(18.03)	0(0.00)	132(1.87)
No	600(81.97)	6318(100.00)	6918(98.13)

^a^ missing information on maternal education (*n* = 13 (0.2%) and anemia (*n* = 33 (0.5%))

### Prevalence of preterm births

A total of 686 babies were considered preterm (prevalence 9.7% (95% CI 9.0–10.4%). Of these, 558 (81%) were identified based on clinical assessment at birth and 128 (19%) based on a birth weight of <2000 g. The distribution of the preterm births were 11% between 24 and 27 weeks of gestation, 22% between 28 and 31 weeks of gestation and 67% between 32 and 36 weeks of gestation. Of the 6364 term births 721(11%), were small for gestational age with weight between 2000 g and 2500 g.

### Performance of the HRPB package to detect preterm births

Of the 7050 women included in this study, 732 (10.4%) were HRPB positive. Of these 732 women, 333 delivered a preterm baby (positive predictive value 48.5%, 95% CI 44.7% – 52.4%). The false negative rate was 5.6% and false positive rate was 54.5%. The frequency and proportion of HRPB symptoms, and the test characteristics for each symptom are described in Table 2. The likelihood ratio associated with being HRPB positive was 8.1 (95% CI 7.2–9.3). As shown in Table 3 the LR of a preterm birth increases with increasing number of signs or symptoms, specifically from a probability of preterm birth in women with no signs or symptoms of 6% to 68% in those who had 3 or more signs or symptoms. Furthermore, the mean birth weight consistently decreased with increasing number of symptoms from a mean of 2704 g (+/− 402) for no signs or symptoms to 2429 g (+/− 549) for 1 sign or symptom, 2308 g (+/− 557) for 2 signs or symptoms and 2187 g (+/− 616) for 3 or more signs or symptoms. The Cochrane-Armitage test for linear trend was highly significant (*p* < 0.0001). In the multivariable GEE analysis, we found that independent of other clinical covariates, the number of high risk symptoms was a statistically significant predictor of preterm births. The odds ratio for preterm birth was 10.3, 19.4 and 37.7 when 1, 2 or 3 or more HRPB signs or symptoms respectively were present.

**Table 2:** Frequency and proportion of HRPB symptoms, and the test characteristics of each symptom

Symptom	N (% out of 7050)	Sensitivity	Specificity	Positive Predictive Value	Negative Predictive Value
Signs of Preterm Labor	540 (7.66)	49.3%	93.5%	38.8%	95.7%
Premature rupture of membrane	251 (3.56)	52.6%	91.9%	19.2%	98.1%
Hemorrhage	71 (1.01)	64.8%	90.8%	6.7%	99.6%
Hypertension	132 (1.87)	37.9%	90.8%	7.3%	98.7%

**Table 3:** Performance of Risk-Stratification as a Predictor of Preterm Birth

Number of HRPB Signs or symptoms	Outcome – Reference Standard (N)		Probability of Delivering Preterm	Likelihood Ratio (95% Confidence Interval)	Odds Ratio from GEE Model (95% Confidence Interval) ^a^
	Preterm	Term			
0	353	5965	0.06	0.55 (0.50–0.60)	Reference
1	173	273	0.39	5.89 (4.44–7.81)	10.3 (8.14–13.0)
2	139	116	0.55	11.05 (7.51–16.27)	19.4 (14.6–25.8)
3 or more	21	10	0.68	19.51 (0.84–454.0)	37.7 (17.4–81.9)
Total	686	6364			

^a^ Results are based on multivariable logistic regression analyses that included the following covariates and was adjusted for inter-cluster differences using GEE: mother’s age, education, parity and anemia

Cochran - Armitage test for linear trend: Χ^2^ (1 df) = 1215.81, *p* = 2.2 × 10^−266^

### Risk stratification and time to delivery and neonatal outcomes

**Fig. 2:**
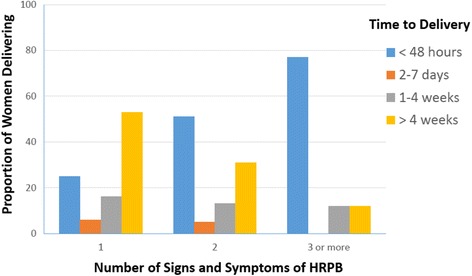
Time to delivery in women at HRPB by number of signs or symptoms

Figure 2 shows that the likelihood of a delivery over time indicating that more signs or symptoms of HRPB was associated with shorter time to delivery and fewer signs or symptoms were associated with longer time to delivery. We found that over 40% of the pregnancies continued for four or more weeks from the time of identification but the probability was only 11% when there were 3 symptoms as compared to 53% when there was only 1 symptom. We also investigated the association of risk stratification with neonatal outcomes. The 7050 women delivered 97 (1.4%) stillbirths, 207 (2.9%) neonates died and 471 (6.7%) babies had at least one postnatal complication (feeding problems, congenital anomalies, breathing problems, high fever, hypothermia and convulsions singly or in various combinations). Table 4 shows the risk of adverse neonatal outcomes using generalized estimating equations that accounted for inter-cluster variations. The risk of stillbirths, postnatal death and postnatal complications was significantly associated with the number of signs or symptoms of HRPB identified by the community health workers and the risk of all neonatal outcomes increased significantly with increased number of signs or symptoms.

**Table 4:** Neonatal Outcomes by Number of Signs or Symptoms of HRPB

Outcome	Number of symptoms			^a^P_trend_
	1	2	3 or more	
Stillbirths	2.64 (1.45–4.80)	3.96 (2.07–7.57)	6.06 (1.42–25.8)	χ^2^ = 32.73, P < 0.001
Postnatal deaths	3.21 (2.17–4.75)	2.25 (1.27–3.98)	4.22 (1.28–14.1)	χ^2^ = 32.93, *P* < 0.001
Postnatal complications	2.56 (1.80–3.63)	2.95 (1.95–4.45)	5.11 (2.16–12.1)	χ^2^ = 87.14, P < 0.001

^a^ Cochrane-Armitage test for linear trend

### Improvement in prediction of a preterm birth using risk stratification

Lastly, we investigated whether the addition of being HRPB positive to a base model that included maternal age and education, parity and anemia improved the prediction of preterm birth. These covariates were chosen on the basis of their significant statistical association with the outcome of preterm labor (Table 1). The IDI value for the addition of being HRPB positive was 16.78% (95% CI 14.93–18.63%) and the NRI was 30.61% (95% CI 25.31–35.91%), both of which are highly significant (*p* < 1.0 × 10^−22^). These indexes thus showed that being HRPB positive clearly improved the prediction of women delivering a preterm baby over and above a baseline risk.

## Discussion

In this secondary analysis of pregnant women in rural communities near Nagpur, Central India who received a package of interventions to detect those at HRPB, we found that identification of any of the 4 specific signs or symptoms of preterm labor was an independent predictor of a subsequent preterm birth. The LR+ were >5 and ranged from 5.9 for presence of 1 symptom to 19.5 for presence of 3 or more symptoms. In addition, the number of signs or symptoms of HRPB directly translated into an increasing risk of preterm birth, a decreasing birth weight, higher likelihood of early delivery and higher risk of adverse neonatal outcomes. The package was easy to use and implement in a rural setting and improved the risk stratification of the pregnant women at HRPB. Honest et al. identified a combination of tests predicting preterm births. Only a few tests had LR+ > 5. In asymptomatic women these were ultrasonographic cervical length measurement and cervicovaginal prolactin and fetal fibronectin screening for predicting spontaneous preterm birth before 34 weeks. In symptomatic women with threatened preterm labour, tests with LR+ > 5 were absence of fetal breathing movements, cervical length and funnelling, amniotic fluid interleukin-6 (IL-6), serum CRP for predicting birth within 2–7 days of testing, and matrix metalloprotease-9, amniotic fluid IL-6, cervicovaginal fetal fibronectin and cervicovaginal human chorionic gonadotrophin (hCG) for predicting birth before 34 or 37 weeks [17]. These investigation are unavailable and not feasible in low resource settings.

The overall prevalence of preterm birth in this community was 10% which is lower than the reported prevalence of 13% preterm births in India [1] Our lower prevalence of preterm birth may be due to recent improvements in community obstetric care, continuum of care and facility based deliveries at our study site [8, 10].

All babies <5th percentile were considered preterm due to lack of reliable ascertainment of GA in rural population. Only 19% of the preterm births were identified based on birth weight. Low Birth Weight (LBW) was used as a proxy for preterm birth because in Global Network sites approximately 15% of the women who delivered at health centers or homes did not know their LMP dates, and 40% of the deliveries occurred at the community level, where ultrasound is not available.

We found that over 40% of the pregnancies continued for four or more weeks from the time of symptom identification. However, when we investigated the distribution of the time interval between risk stratification and delivery based on the gestational age at which women were enrolled, we observed that those enrolled early during pregnancy (e.g. 24–28 weeks) were more likely to continue the pregnancy for ≥4 weeks as compared to women who were enrolled later on (Fig. 2). Considering that most of the women with only one risk symptom were able to continue pregnancy for ≥4 weeks beyond risk stratification, it is conceivable that the package of interventions may lend itself to a lead-time bias [18]. It is possible, for example, that the package of intervention may be more accurate in risk stratifying women closer to term than in earlier during pregnancy. Consequently, the optimum time point in pregnancy at which the package of intervention will be most beneficial in community settings is currently unknown. More studies are required to address this important operational issue.

Our study has some limitations that were imposed by ACT trial protocol. First, the inclusion and exclusion criteria for the trial may make our study population different from many rural populations. Secondly, ultrasound corroboration of gestational age was rarely available. Third our reference standard to define preterm birth based on clinical evaluation and birth weight may have resulted in misclassification of preterm birth. The magnitude and direction of the influence of misclassification on the performance of the package of intervention is not known. We did try to address this by running sensitivity analyses to model the influence of errors in reference standard on the estimates of likelihood ratios of being HRPB positive [19]. Figure 1 shows that even if the reference standard were to inflate the prevalence of preterm birth by 10%, the estimated likelihood ratio for being HRPB positive would fall from 8.1 (as reported in this study) to 7.6 – a negligible decrease. Fourth, this was a symptom based intervention to identify women at HRPB. It did not include clinical history such as previous preterm birth which may improve the predictability of a preterm birth. Fifth, as discussed above the package of intervention may be more accurate in risk stratifying women closer to term than in earlier during pregnancy. Sixth, if a woman at HRPB did not deliver within a short time of onset of symptoms, there was no way to know whether the baby was actually premature at the time of onset of symptoms, and perhaps reduced the predictability of the HRPB intervention package. Finally, we have not studied the potential cost-effectiveness analysis of the intervention package of intervention. If this approach is found to be successful in other populations, costs of training community health workers, costs of false positive identification, etc. would need to be considered.

## Conclusions

Currently in the rural areas of developing countries there are no reliable methods to detect at risk mothers for preterm deliveries. To our knowledge, this is the first, large community based study that has evaluated from a pragmatic standpoint a easy to use package of interventions to identify and risk stratify women at HRPB. The HRPB package of interventions was found to be useful where the community health workers who are the first point of contact for most rural mothers provide a strong link between communities and the health system. This intervention could help to facilitate the administration of antenatal corticosteroids, but could be adapted to other interventions to improve outcomes in preterm babies. Further studies are needed to refine this tool and investigate its validity and acceptability in other populations with varying prevalence of preterm births and, different health care infrastructures. Furthermore the cost effectiveness in reducing the burden of mortality due to prematurity needs to be determined before it is adopted for use in the public health settings.

## Abbreviations

ACT: Antenatal Corticosteroid Trial
ASHA: Accredited Social Health Activist
DALYs: Disability Adjusted Life Years
GEE: Generalized Estimating Equations
HCP: Health Care Provider
HRPB: High Risk for Preterm Birth
IDI: Integrated Discrimination Improvement
IRB: Institutional Review Board
JSY: Jannani Suraksha Yojana
LRs: Likelihood Ratios
MNHR: Global Network Maternal Newborn Health Registry
NICHD: Eunice Kennedy Shriver National Institute of Child Health and Human Development
NRI: Net Reclassification Index
PHC: Primary Health Center
RDS: Respiratory Distress Syndrome
SC: Subcenter
